# Targeted population genomics uncovers demographic history and genetic divergence in north American wild cranberry

**DOI:** 10.1093/hr/uhag161

**Published:** 2026-04-21

**Authors:** Nicolás Pandeli Jiménez, Maria Alejandra Torres-Meraz, Jeffrey L Neyhart, Juan Zalapa, Gina Maria Sideli

**Affiliations:** P.E. Marucci Center for Blueberry and Cranberry Research and Extension, Rutgers, The State University of New Jersey, Chatsworth, NJ 08019, USA; Department of Plant and Agroecosystem Sciences, University of Wisconsin-Madison, Madison, WI 53706, USA; USDA, Agricultural Research Service, Genetic Improvement for Fruits and Vegetables Laboratory, Chatsworth, NJ 08019, USA; Department of Plant and Agroecosystem Sciences, University of Wisconsin-Madison, Madison, WI 53706, USA; United States Department of Agriculture-Agricultural Research Service, Vegetable Crops Research Unit, Madison, WI 53706, USA; P.E. Marucci Center for Blueberry and Cranberry Research and Extension, Rutgers, The State University of New Jersey, Chatsworth, NJ 08019, USA

## Abstract

Wild populations of North American cranberry (*Vaccinium macrocarpon* Aiton) are reservoirs of genetic variation that may contribute to the improvement of breeding-relevant traits. However, the extent to which wild genetic variation is geographically structured and represented in elite germplasm remains unclear. We analysed 179 wild cranberry accessions from the upper Midwest and Eastern North America to estimate nucleotide diversity (*π*), population structure, and loci associated with genetic differentiation and environmental variables using a genome-informed targeted genotyping panel. Additionally, 14 demographic scenarios were evaluated using site-frequency-spectrum-based inference to identify historical events that could explain current genetic diversity. We observed extremely low nucleotide diversity within the targeted panel (*π* = 5 × 10^−6^). Rare allele distributions strongly influenced *π* and Tajima’s *D* values, suggesting constrained diversity in the genomic regions assayed that is not captured by heterozygosity-based estimates alone. However, we interpreted these results as conservative lower bounds on genome-wide neutral diversity because the targeted panel is enriched for genic and conserved regions. A clear separation between the Midwest and East populations was observed, with inbreeding coefficients ranging from −0.13 to 0.15. Furthermore, site frequency spectrum inference from the targeted panel supported a demographic scenario consistent with a significant population reduction ≈15–14 thousand years ago (kya), followed by a divergence between the two regions ≈12 kya, and an asymmetric gene flow ≈1.3 kya. We detected 254 candidate loci showing regional allele-frequency differentiation. Several of these loci colocalized with candidate genes linked to stress response, development, and metabolic processes. To evaluate the representation of geographically differentiated wild alleles in a breeding context, we analysed Rutgers breeding materials (*n* = 484) and found that this panel is enriched for common alleles in Eastern wild populations. These findings indicate regionally structured allele-frequency variation in wild cranberry, with potential relevance to environmental response and breeding. This study extends prior wild cranberry population-genetic research by providing targeted-panel estimates of diversity, comparisons of demographic models, and breeding insights on geographically differentiated alleles, while highlighting the importance of conserving wild cranberry germplasm for use in modern breeding programs.

## Introduction

The domestication process of crops together with the subsequent improvement of traits leads to a reduced genetic diversity and selection response in breeding programs, causing genetic bottlenecks and requiring the introduction of genetic variation from new sources [1–3]. Crop wild relatives (CWRs) are genetic reservoirs of novel alleles that could improve agronomic and consumer preference traits in economically relevant crops [4, 5]. For example, the introduction of wild alleles into cultivated backgrounds has shown a substantial contribution to improving traits such as flavor [6], yield [7], and biotic [8] and abiotic [9] stress tolerance. Therefore, the discovery and targeted transfer of CWR alleles provides a breeding strategy to increase genetic gain in breeding programs.

North American cranberry (*Vaccinium macrocarpon* Aiton) is a high-value fruit crop recognized for its abundant antioxidant compounds that promote human health benefits [10–12]. In 2024, 37 000 acres of cranberries were harvested in the USA, resulting in a total production value of 339.59 million US dollars [13]. However, this milestone has not come without cost, as growers constantly face multiple production difficulties. As seasonal temperatures fluctuate during the cranberry growing period, farmers must control insects and diseases while protecting their crops from extreme heat and cold temperatures to maintain production yields. Among the most challenging problems are complex fruit rot, caused by multiple pathogens [14, 15], and the re-emergence of false-blossom disease, transmitted by the blunt-nose leafhopper [16, 17], which can be a significant threat to cranberry production if it continues to spread across growing regions. Over ~200 years, cranberry breeders have achieved high-yielding cultivars with larger berries; however, several other economically relevant traits have shown minimal differences between wild and cultivated materials [18, 19]. For example, Jiménez *et al.* [19] found no significant differences between wild, landrace, and breeding materials for the flavonol content of cranberry fruit. However, several wild and landrace accessions outperformed breeding lines. Furthermore, genome-wide associations for environmental conditions conducted by Neyhart *et al.* [20] and for flavonols performed by Jiménez *et al.* [19] identified several segregated loci in wild cranberry populations. From these studies, it is clear that adaptive and quality CWR alleles should be exploited to widen the genetics and broaden the response to selection in cranberry breeding programs.

Genetic diversity in wild cranberry has been a central focus of discussion across studies, ranging from extremely low in early allozyme surveys [21] to moderate-high in later simple sequence repeat (SSR) and single nucleotide polymorphism (SNP) studies using heterozygosity estimates [20, 22–25]. Although heterozygosity estimates have been widely reported in wild cranberry, no studies have explored cranberry diversity based on nucleotide diversity (*π*). The key difference between *π* and heterozygosity is that *π* summarizes sequence variation across surveyed nucleotide sites, providing a complement to heterozygosity by capturing sequence-level variability rather than allelic variation at a specific site [26]. Therefore, *π* can provide additional information on genetic diversity by revealing genomic signatures shaped by demographic history and selection in native populations. For a better characterization of the genetic diversity derived from heterozygosity and *π*, Tajima’s *D* tests whether the diversity pattern in a natural population is unexpected and may be due to deviation signals from neutral equilibrium driven by bottlenecks, expansions, or structures [27]. Bruederle *et al.* [21] inferred from allozyme data using the Nei method [28] that a severe Pleistocene bottleneck ≈110 thousand years ago (kya) reduced the genetic diversity of wild cranberry. To date, this is the only study that has attempted to link historical events to cranberry genetic diversity and remains untested through simulation-based demographic inference [29–31]. Furthermore, a single-bottleneck scenario does not explain other processes, including range-wide wild cranberry population splits across North America, which can also influence current diversity patterns. With the intent of making further advancements in cranberry breeding, it is clear that more analyses are necessary to uncover historical events and evaluate the starting baseline diversity in wild cranberry.

Here, our study assembled a wild cranberry collection from locations in the upper Midwest and Eastern North America to characterize genomic variation using diversity metrics, including *π*, heterozygosity, inbreeding coefficient (*G*_IS_), and allele-frequency spectrum analyses based on the *Vaccinium* pangenome-derived targeted genotyping platform focused on genic loci from core and accessory genes and QTL-linked regions. We studied population structure and broad-scale genomic differentiation in genotypes collected from nine US states and one Canadian province. We also compared alternative demographic models to infer historical events that may help explain the genetic diversity observed in wild cranberry. In addition, we conducted genome-wide association analyses to detect genomic regions and candidate genes related to population differentiation patterns, geographic structure, and environmental conditions. This study represents the first population-genomic analysis of wild cranberry using markers derived from the *Vaccinium* pangenome and provides evolutionary inference from targeted functional genomic regions. These findings establish a historical and allelic context to guide the introduction of geographically differentiated CWR alleles into cranberry breeding programs.

## Results

### Population-level genetic diversity and differentiation patterns

**Figure 1:**
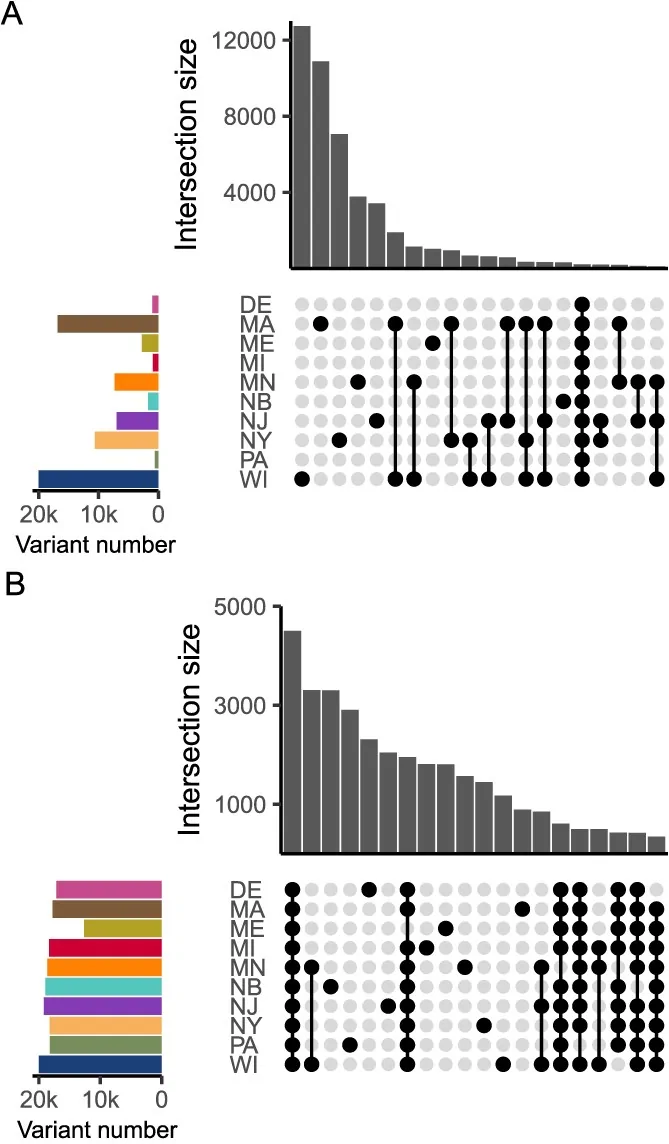
Intersection of DNA variants among North American wild cranberry populations. Visualization of shared (A) rare (0.01 ≤ MAF ≤ 0.05) and (B) common (MAF > 0.5) DNA variants between Delaware (DE), Massachusetts (MA), Maine (ME), Michigan (MI), Minnesota (MN), New Brunswick (NB), New Jersey (NJ), New York (NY), Pennsylvania (PA) and Wisconsin (WI) populations. The bottom-left bar plot provides the total number of SNPs and INDELs detected within each population. The top-right bar plot displays the size of every intersection among populations that share a given set of variants, while the lower-right dot matrix beneath indicates which populations participate in each intersection (black and gray circles indicate population present and absent, respectively).

We studied 10 wild cranberry populations collected in the USA and Canada (Delaware (DE), Massachusetts (MA), Maine (ME), Michigan (MI), Minnesota (MN), New Brunswick (NB), New Jersey (NJ), New York (NY), Pennsylvania (PA), and Wisconsin (WI); Supplementary Data Table S1) whose sample sizes were between 4 (NB) and 45 (MA) accessions (Table 1). Our research identified 19 983 (ME) to 40 690 (MA) DNA variants per population (Table 1, Fig. 1). Most of the detected variants were common (minor allele frequency (MAF) > 0.05), representing at least 60% of the total variants. Rare alleles varied between populations, ranging from <10% (DE, MI, NB, and PA) to >40% (MA and WI) of all variants. More than 80% of the DNA variants were SNPs (Table 1) with a collection-wide mean MAF of 0.18 ± 0.15 (Supplementary Data Fig. S1) and population-specific means ranging from 0.15 (MA and WI) to 0.33 (NB; Table 1). Interestingly, the insertion–deletion (INDEL):SNP variant ratio showed a relative enrichment of INDELs among rare variants compared with common variants. The common INDEL:SNP ratios remained between 8 and 14% between populations, while the rare INDEL:SNP ratios ranged between 12 and 45%. MI (37%), NY (43%), and PA (45%) populations showed a high rare INDEL:SNP ratio. In contrast, ME, MN, and WI showed lower rare INDEL:SNP ratios with proportions of 16, 12, and 15%, respectively.

**Table 1:** Population-level inbreeding and allele frequncy spectrum in 10 wild cranberry populations across Midwest and Eastern North America. Wild cranberry accessions were collected from nine US states and one Canadian province, including Delaware (DE), Massachusetts (MA), Maine (ME), Michigan (MI), Minnesota (MN), New Brunswick (NB), New Jersey (NJ), New York (NY), Pennsylvania (PA), and Wisconsin (WI). Rare and common SNPs and INDELs were identified using MAF thresholds of 0.01 ≤ MAF < 0.05 and MAF > 0.05, respectively. The inbreeding coefficient (*G*_IS_), observed heterozygosity (*H*_O_), and expected heterozygosity (*H*_E_) were calculated for each population.

			**Rare DNA variants**				**Common DNA variants**						
Population	*n* ^a^	MAF^b^	SNP	INDEL	Private (%)^c^	Overlap (%)^d^	SNP	INDEL	Private (%)	Overlap (%)	*G* _IS_	*H* _O_	*H* _E_
DE	9	*0.25± 0.14*	772	216	8.6	21.3	21 119	2400	9.8	19.2	0.03	0.27	0.28
MA	45	*0.15± 0.15*	13 599	2751	66.5	1.3	22 444	1896	3.7	18.5	0.10	0.26	0.29
ME	5	*0.27± 0.13*	2292	371	38.8	7.9	15 369	1951	10.4	26.0	0.09	0.21	0.23
MI	10	*0.26± 0.13*	642	240	3.3	23.8	22 849	2316	7.2	17.9	0.01	0.30	0.31
MN	19	*0.21± 0.15*	6343	773	53.0	3.0	23 415	2112	6.1	17.7	0.11	0.21	0.24
NB	4	*0.34± 0.11*	1346	320	19.1	12.6	22 729	3267	12.7	17.3	−0.13	0.35	0.31
NJ	15	*0.22± 0.14*	5702	1056	50.6	3.1	24 054	2271	7.8	17.1	0.05	0.27	0.29
NY	20	*0.21± 0.15*	7238	3077	68.5	2.0	22 777	2261	5.8	18.0	0.03	0.30	0.31
PA	8	*0.25± 0.13*	410	184	4.4	35.4	22 697	2262	11.6	18.1	0.04	0.31	0.32
WI	44	*0.15± 0.15*	16 900	2496	65.7	1.1	25 438	2001	4.3	16.4	0.15	0.23	0.28

a Number of accessions sampled in each US state or Canadian province.

b Average and standard deviation of MAF in each population, including rare and common SNPs.

c Percentage of unique DNA variants in each population.

d Percentage of shared DNA variants across the collection.

The overlap of DNA variants between wild populations revealed that common genetic variants spread more widely throughout the entire dataset (Fig. 1B) than rare variants (Fig. 1A). The common variants shared among the populations ranged from 17 to 26% (Table 1), where the ME population showed the highest overlap. However, the distribution of rare variants showed a more widespread range, from 1 to 35% (Table 1); the DE, MI, and PA populations reported the highest overlap of rare genetic variants in all wild populations. We noticed that rare private variants exceeded common private variants (Table 1). Private common variants comprised 3.7–12.7% of each population’s total genetic makeup. In contrast, rare private variants ranged from 3.3 to 68.5%, with values of >50% in the MA, MN, NJ, NY, and WI populations (Table 1).

SNPs with MAF ≥ 0.01 were broadly distributed across all 12 cranberry chromosomes (Supplementary Data Fig. S2) and exhibited a highly skewed inter-marker distance distribution, with a median and a much larger mean spacing of 30 bp and 6.4 kb (interquartile range (IQR): 9–210 bp; 5–95% range: 2–40 kb), respectively. Only 3.69% of inter-marker gaps were ≥50 kb, and 1.02% were ≥100 kb. Furthermore, our analysis indicated that 64.67% of the SNPs were located within annotated gene models.

**Figure 2:**
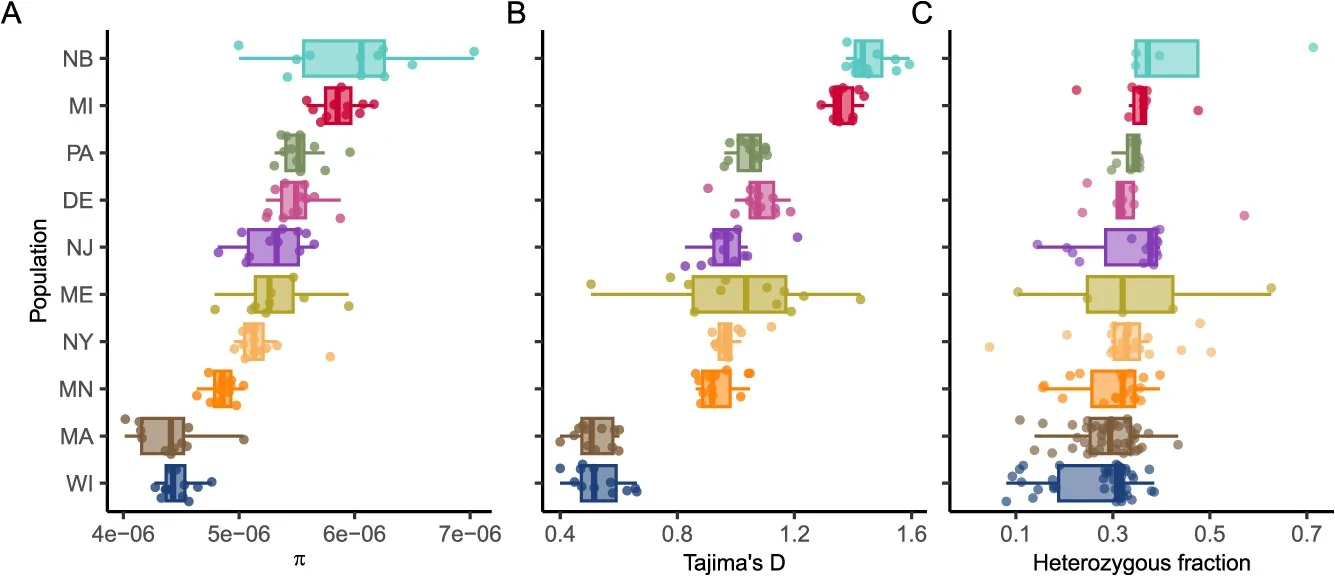
Panel-wide diversity and individual heterozygosity in wild cranberry populations. Box plots of (A) nucleotide diversity (*π*) and (B) Tajima’s *D* estimates in 100-kb windows across targeted regions using SNPs with MAF ≥ 0.01 detected from Delaware (DE), Massachusetts (MA), Maine (ME), Michigan (MI), Minnesota (MN), New Brunswick (NB), New Jersey (NJ), New York (NY), Pennsylvania (PA), and Wisconsin (WI) populations. (C) Box plot of individual heterozygosity for each population calculated from SNPs with MAF ≥ 0.05.

The overall *π* of 5 × 10^−6^ ± 2.2 × 10^−6^ indicated a low genetic diversity in this species. However, most wild populations showed an extensive *π* range (2.6 × 10^−7^ to 2.1 × 10^−5^) except ME and NB (3.5 × 10^−6^ to 9.0 × 10^−6^; Supplementary Data Fig. S3). The observed *π* range among populations translated into differences in genetic variability at the population level (Fig. 2A). For example, the MA (*π̅* = 4.5 × 10^−6^) and WI (*π̅ =* 4.5 × 10^−6^) populations displayed low genetic diversity compared with the overall mean, while the MI (*π̅* = 5.9 × 10^−6^) and NB (*π̅* = 6.1 × 10^−6^) populations exhibited higher genetic variability. Additionally, our overall Tajima’s *D* (0.89 ± 0.86) detected a departure from neutrality in the collection, where some genomic regions harbored an excess of rare or intermediate-frequency alleles (*D*_min–max_ = −1.9–3.5). Thus, Tajima’s *D* showed different levels of deviation from neutral equilibrium between wild populations (Fig. 2B). The MA and WI populations showed a slight deviation from the mutation-drift equilibrium (*D̅* = 0.5), while DE, ME, MN, NJ, NY, and PA exhibited moderate positive values (*D̅* = 0.9–1.1). In the case of MI and NB, a stronger shift from neutrality was observed (*D̅* = 1.36 and 1.45, respectively).

The observed heterozygosity (*H*_O_) ranged from 0.21 (MN and ME) to 0.35 (NB), with a mean of 0.27, while the expected heterozygosity (*H*_E_) ranged from 0.23 (ME) to 0.32 (PA), with a mean of 0.29. Additionally, the collection showed an average heterozygosity fraction per individual of 0.30 ± 0.09. The values of the heterozygosity fraction within the populations ranged from 0.27 (WI) to 0.45 (NB), where the populations showed narrower IQR than others (Fig. 2C). For example, the DE (IQR = 0.03), MI (IQR = 0.02), and PA (IQR = 0.02) populations showed highly homogeneous heterozygosity levels among the accessions sampled. In contrast, the heterozygosity levels of ME (IQR = 0.18), NB (IQR = 0.13), and WI (IQR = 0.13) suggested greater heterozygosity variation between accessions. Furthermore, the inbreeding coefficient (*G*_IS_) in the wild collection indicated a spectrum of excess heterozygous to moderate heterozygous deficit (Table 1). The estimates of *G*_IS_ for the DE, MI, NJ, NY, and PA populations were near zero (*G*_IS_ = 0.01–0.05), while the MA, ME, MN, and WI populations showed slightly positive values (*G*_IS_ = 0.09–0.15). The NB population stood out as the only one with a negative coefficient (*G*_IS_ = −0.13).

### Analysis of population structure and admixture composition in the collection

**Figure 3:**
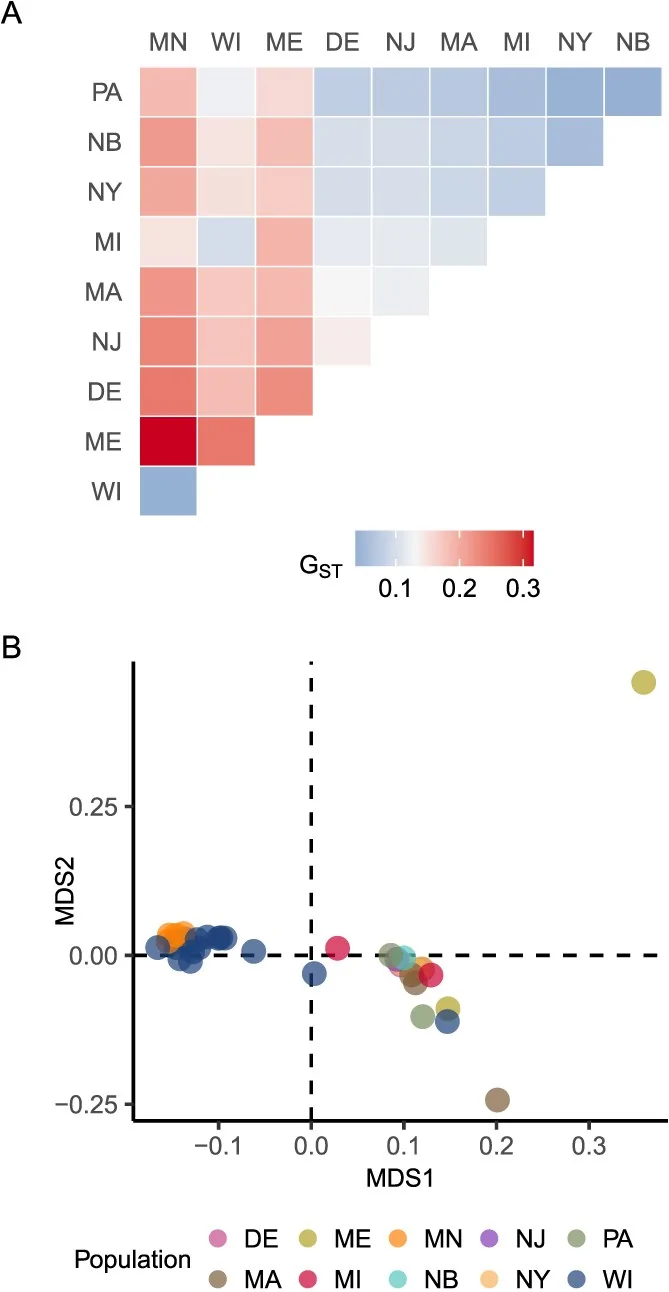
Population differentiation and spatial genetic structure in wild cranberry populations. (A) Heat map shows pairwise comparison of genetic differentiation (*G*_ST_) between Delaware (DE), Massachusetts (MA), Maine (ME), Michigan (MI), Minnesota (MN), New Brunswick (NB), New Jersey (NJ), New York (NY), Pennsylvania (PA), and Wisconsin (WI) populations, while (B) MDS analysis indicates the genetic distance based on *G*_ST_ values for the collection sites in the nine US states and one Canadian province.

**Figure 4:**
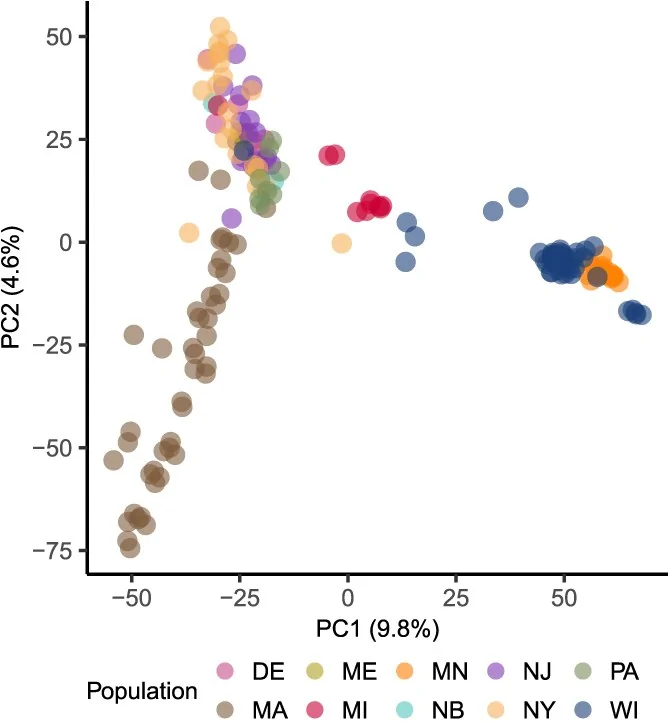
. Population structure among North American wild cranberry populations. PCA of wild cranberry populations collected from Delaware (DE), Massachusetts (MA), Maine (ME), Michigan (MI), Minnesota (MN), New Brunswick (NB), New Jersey (NJ), New York (NY), Pennsylvania (PA), and Wisconsin (WI). PCA was performed with 16 070 SNPs mapped on the reference genome ‘Ben Lear’ v1.0.

The extensive sharing of common genetic variants among wild populations (Fig. 1B), predominantly SNPs (Table 1), was reflected in the moderate level of genetic differentiation detected in this collection (*G̅*_ST_ = 0.14). However, the analysis of *G*_ST_ in wild populations showed distinct differentiation patterns through pairwise *G*_ST_ values (Fig. 3A). The genetic differences between MN and WI were minimal (*G*_ST_ = 0.04), while the *G*_ST_ values between DE, MA, MI, NB, NJ, NY, and PA indicated moderate genetic differentiation (*G̅*_ST_ = 0.14; *G*_STmin–max_ = 0.04–0.14). ME showed the highest level of differentiation from all other groups, with an average *G*_ST_ of 0.21 (*G*_STmin–max_ = 0.16–0.32). Furthermore, multidimensional scaling (MDS) of the genetic distance based on *G*_ST_ pairwise comparison between collection sites revealed spatial genetic differentiation in wild populations (Fig. 3B). The first coordinate (MDS1) distinguished the wild accessions of MN and WI on the one side of PC1 (Fig. 3B) from those collected in DE, MA, ME, MI, NB, NJ, NY, and PA on the the other side of PC1 (Fig. 3B) The results of our principal component analysis (PCA) showed similar population stratification patterns observed in the MDS analysis (Fig. 4). The right side of the PC1 axis separated the MN and WI populations from the DE, MA, ME, NB, NJ, NY, and PA populations on the left side. Interestingly, the MI population was located between the two groups, while wild accessions from MA showed a wide genetic differentiation across PC2.

**Figure 5:**
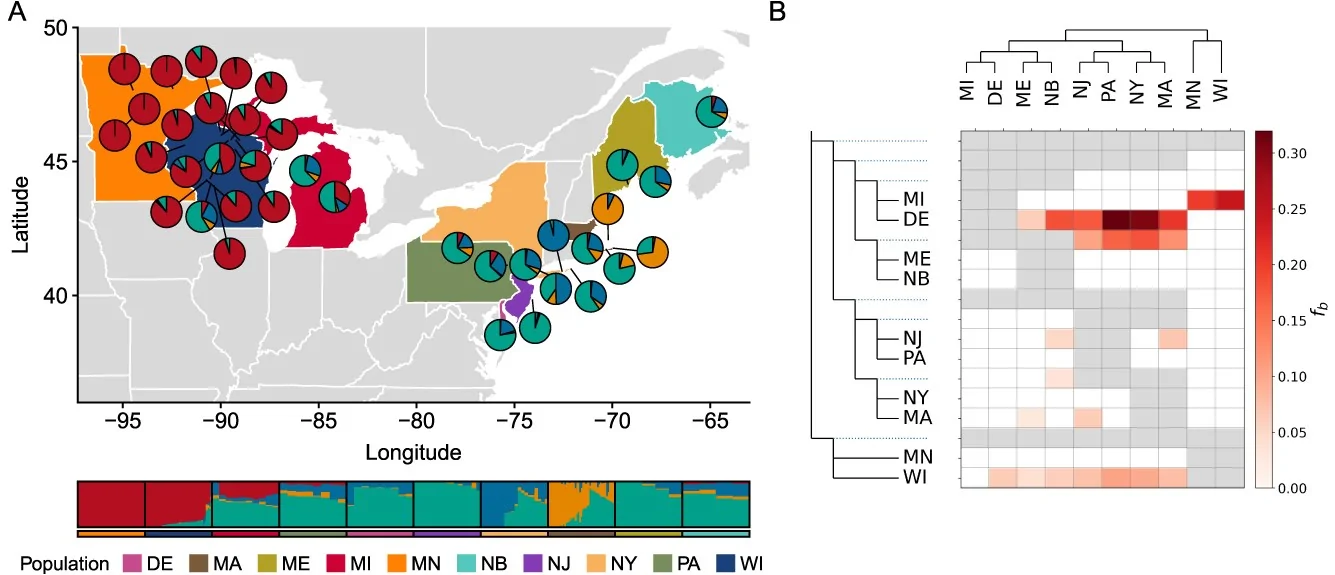
Geographic distribution and introgression signals of ancestral genetic clusters in North American wild cranberry populations. (A) The geographic map displays pie charts which show ancestry proportions across collection sites of wild cranberry populations sampled in Delaware (DE), Massachusetts (MA), Maine (ME), Michigan (MI), Minnesota (MN), New Brunswick (NB), New Jersey (NJ), New York (NY), Pennsylvania (PA), and Wisconsin (WI). The vertical bar below represents individual-level admixture proportions for each accession. The four ancestral genetic clusters identified at the optimal admixture level (*K* = 4) are represented by red (C1), blue (C2), yellow (C3), and turquoise (C4) colors. (B) The heat map reveals introgression detected with the Fbranch statistic (*f*_b_), which assigns gene flow signals to particular clades of the tree. The *Y*-axis shows the population-level phylogeny rooted with *Vaccinium oxycoccos* (outgroup). Darker red corresponds to higher *f*_b_ values (0–1), while gray indicates the absence of introgression. Blue dotted lines indicate internal nodes representing most recent common ancestors.

We evaluated the composition of ancestry in the collection by estimating the proportions of admixture across cluster numbers (*K*) from 1 to 10 (Supplementary Data Fig. S4, Fig. 5A). The analysis showed that *K* = 4 represented the optimal level of admixture because increasing *K* beyond this point produced only a small decrease in cross-validation error (Supplementary Data Fig. S5). The admixture analysis revealed specific ancestral patterns aligned with the geographic distribution of wild populations (Fig. 5A). The ancestry of the MN and WI populations consisted mainly of the ancestral cluster C1 with representations of 99.9 and 81.7%, respectively. Although the WI population was predominantly C1, a wide range of variations within the population was observed in the ancestral groups C2 (0.0–25.5%), C3 (0.0–12.9%), and C4 (0.0–58. 8%). In contrast, Eastern populations showed predominantly C4 ancestry with high percentages found in DE (77.7%), MA (41.4%), ME (79.2%), NB (67.3%), NJ (94.8%), NY (42.1%), and PA (64.4%). However, these Eastern populations also showed considerable admixture, reflecting varied ancestral backgrounds. For example, the NY population showed a unique genetic profile through its combination of ancestral clusters of C2 (51. 6%) and C4 (42. 1%). Similarly, the MA population showed a different genetic make-up because its ancestry consisted mainly of C3 (48.1%) and C4 (41.4%). The MI population located between homogeneous Midwest populations (MN and WI) and admixed Eastern populations showed an intermediate genetic makeup (C1 = 18.7%, C2 = 19.3%, C3 = 5%, and C4 = 57%). To reinforce the admixture evidence, a neighbor-joining phylogenetic tree was generated (Supplementary Data Fig. S6). The resulting topology reflected the geographic clustering, revealing two major clades that corresponded to the Midwest and Eastern populations. Most of the MI accessions appeared intermediate between the two groups; in particular, two clustered with the Eastern clade, consistent with their elevated ancestry of C2 (25 and 27%, respectively).

### Demographic history and gene flow patterns among Midwest and Eastern populations

**Figure 6:**
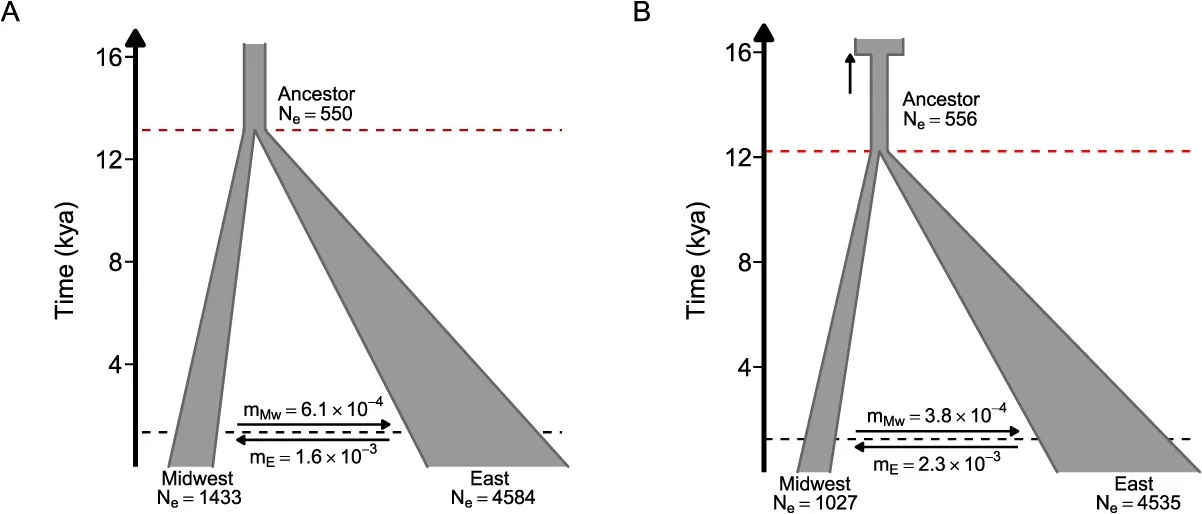
Inference of historical events underlying the genetic divergence between Midwest and Eastern North American wild cranberry populations. Visualization of the best two demographic models (I and M) that explain the genetic divergence between Midwest and Eastern wild cranberry populations. (A) Model I indicates that the Midwest and Eastern populations diverged from their common ancestor ≈13.1 kya (red dashed line) before recent bidirectional migration began ≈1.3 kya (black dashed line). (B) Model M shows that the ancestral population experienced a historical bottleneck event between ≈15.9 and 14.4 kya (black arrow) before splitting into Midwest and Eastern populations at ≈12.2 kya (red dashed line). Model M describes that recent gene flow began ≈1.3 kya (black dashed line). The Midwest population included samples from Michigan (MI), Minnesota (MN), and Wisconsin (WI), while the Eastern population included samples from Delaware (DE), Massachusetts (MA), Maine (ME), New Brunswick (NB), New Jersey (NJ), New York (NY), and Pennsylvania (PA). The models display effective population size (*N*_e_) for diploid individuals and migration rates between west and east (m_Mw_ and m_E_) directions. A generation time of 4 years was assumed for demographic modeling.

We analysed 14 demographic models (Supplementary Data Fig. S7) to understand the historical events that led to the genetic divergence between wild cranberry populations in the Midwest and Eastern North America (Figs 3–5A). The best supported model based on the Akaike information criterion (AIC) was Model I, which described an initial population split followed by contemporary gene flow (AIC = 22 755; Table 2). Model I estimated that these two populations separated ≈13.1 ± 0.12 kya and started to experience gene flow ≈1.3 ± 0.01 kya. Most of the genetic flow occurred from the East to the Midwest rather than from the Midwest to the East (1.9 ± 0.23 and 0.2 ± 0.04 diploids per generation, respectively; Fig. 6A); the Eastern population sustained an effective population size (*N*_e_) 3.2 times larger than the Midwest population (Fig. 6A). However, we noticed that Model M, which incorporated a bottleneck event before the Midwest–East divergence, showed the same estimated maximum likelihood and a minimal statistical difference compared with Model I (*Δ*AIC = 1; Table 2, Supplementary Data Fig. S8). The AIC weight of Model M (*ω* = 0.32) demonstrated strong support relative to Model I (*ω* = 0.68). In this case, the ancestral cranberry population in Model M went through a historical bottleneck event with an intensity of 1.9 ± 2 × 10^−4^ between ≈15.9 ± 2.88 and 14.4 ± 0.23 kya that reduced its population size before the population split into East and Midwest populations ≈12.2 ± 0.12 kya (Fig. 6B). Furthermore, *N*_e_ estimates indicated that the size of the East population was 4.4 times larger than that of the Midwest population, while asymmetric recent migration events (≈1.3 ± 0.17 kya) showed that migration from the East to the Midwest was 2.6 ± 1.13 diploids per generation and from the Midwest to the East 0.1 ± 0.08 diploids per generation (Fig. 6B).

**Table 2:** Statistics of the demographic models proposed for the genetic divergence between Midwest and Eastern North American wild cranberry populations. Statistical summary of 14 demographic models analysed in this study (Supplementary Data Fig. S7), including maximum likelihood from the best run, the number of estimated parameters, the mean AIC score across 100 independent runs with the best parameter values, and the *Δ*AIC relative to the top-ranked model (Model I)

Model	Maximum likelihood	Parameter number	AIC	*Δ*AIC
A	−6235	4	28 805	6050
B	−4958	6	22 848	93
C	−7600	5	34 679	11 924
D	−5139	7	23 689	934
E	−6232	7	28 808	6053
F	−4957	9	22 852	97
G	−7488	8	34 726	11 971
H	−5115	10	23 593	838
I	−4937	7	22 755	0
J	−5554	7	25 614	2859
K	−5113	5	23 563	808
L	−4964	5	22 878	123
M	−4937	10	22 756	1
N	−5360	10	24 718	1963

To further investigate the signals of gene flow inferred by demographic models (Fig. 6), we tested allele sharing and branch-specific introgression statistics based on the ABBA-BABA method. Significant signals (*Z*-score >3, *P* < 0.05) revealed variable levels of introgression, ranging from ≈4% between WI and ME (*f*_4_ ratio = 0.04) to ≈40% between ME and PA (*f*_4_ ratio = 0.40) (Supplementary Data Table S2). High levels of introgression were detected among the Midwest populations, where *f*_4_ ratios ranged from ≈18 to 24%. In contrast, Eastern populations showed a broader range of introgression, ranging from ≈4 to 40%. Additionally, weaker signals of gene flow were detected between the Midwest and Eastern locations, though all remained below 9%. Since multiple introgression events may have shaped the population histories, we applied the *f*-branch statistic (*f*_b_) to resolve overlapping *f*_4_ ratio signals, assigning them to specific branches of the population-level tree (Fig. 5B). The most pronounced gene flow was detected between DE and other Eastern locations (particularly DE–PA and DE–NY), indicating strong intra-regional introgression. Within the Midwest, the MI–WI and MI–MN pairs shared the highest number of alleles, emphasizing the ‘genetic corridor’ role of MI in the group. Interestingly, WI was the only Midwest population that showed moderate connections to Eastern populations, although the signals were substantially weaker. In general, the *f*_b_ analysis reflected elevated *f*_4_ ratios in agreement with the admixture (Fig. 5A) and the results of the population structure (Figs 3 and 4).

Finally, to evaluate the role of hydrological geography in shaping gene flow, we grouped populations according to the US Major River basin system [32]. Individuals were assigned to five categories – Midwest Atlantic, New England, Souris–Red–Rainy, Great Lakes, and Upper Mississippi – based on their geographic distribution (Supplementary Data Fig. S8A, Supplementary Data Table S1). Dsuite analysis revealed substantial variation in the levels of introgression, ranging from ≈5 to 56% (*Z* score >3, *P* < 0.05) (Supplementary Data Table S3). The strongest signals were detected between the New England–Mid Atlantic (*f*_b_ = 0.56) and Great Lakes–Upper Mississippi (*f*_b_ = 0.19) basins, corresponding to the Eastern and Midwest groups, respectively (Supplementary Data Fig. S8B). In contrast, the introgression between basins in the Midwest and Eastern regions was relatively low, with *f*_b_ ranging from 0.04 (Upper Mississippi–New England) to 0.09 (Upper Mississippi–Mid Atlantic).

### Identification of outlier loci associated with genetic differentiation and geographic–environmental factors

**Figure 7:**
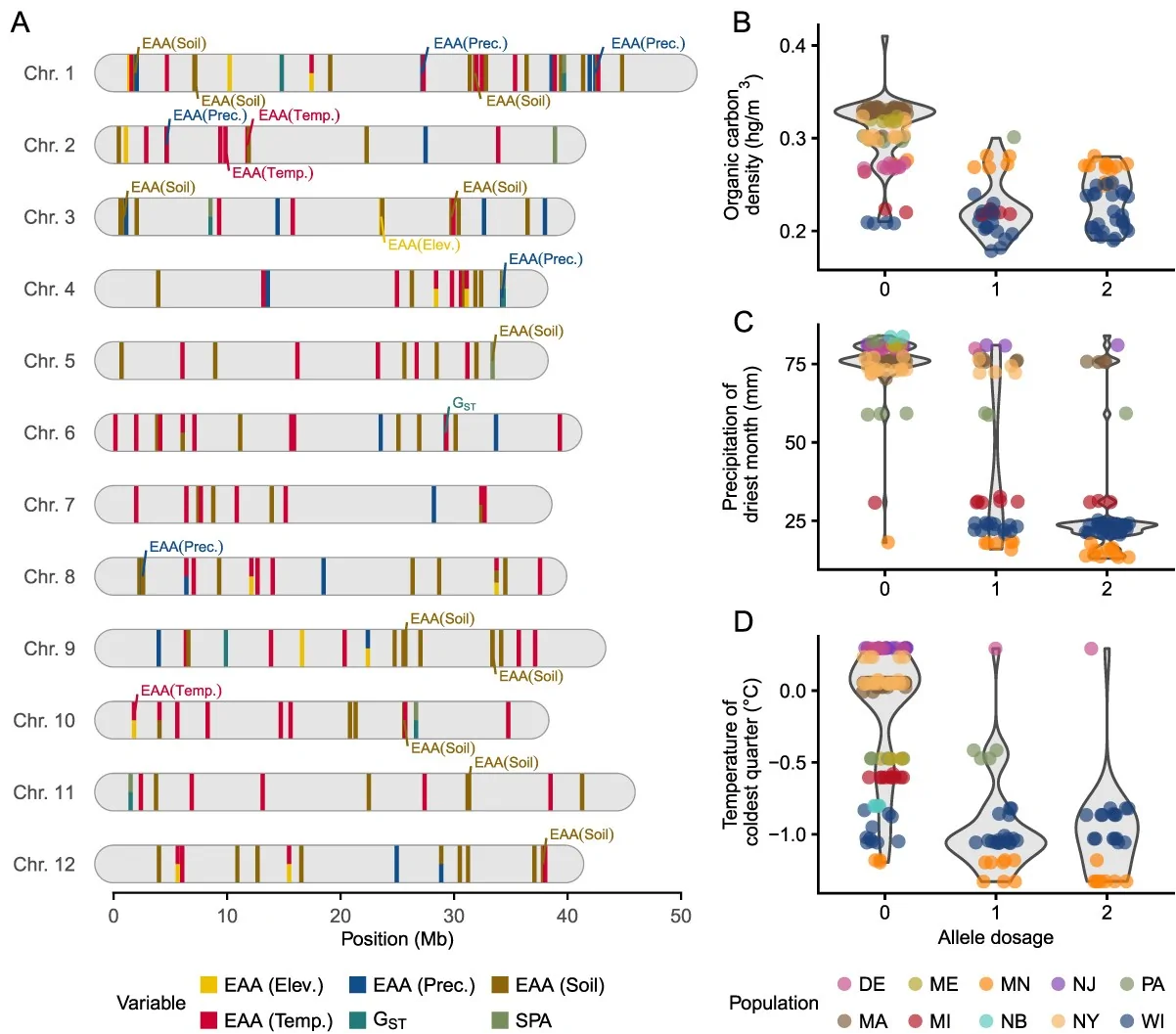
Identification of genomic regions associated with genetic and geographical differentiation in wild cranberry populations. (A) Chromosome-level association plot showing candidate loci identified by environmental association analysis (EAA) for geographic, precipitation, soil, and temperature factors, population differentiation (*G*_ST_), and SPA. (B–D) Boxplots of allele dosage for loci significantly associated with (B) organic carbon density at 5–15 cm soil depth (Vmac_chr05_33388302), (C) precipitation of the driest month (Vmac_chr06_33703561), and (D) temperature of the coldest quarter (Vmac_chr10_15584947) across populations located in Delaware (DE), Massachusetts (MA), Maine (ME), Michigan (MI), Minnesota (MN), New Brunswick (NB), New Jersey (NJ), New York (NY), Pennsylvania (PA), and Wisconsin (WI). Allele dosage values 0, 1, and 2 represent homozygous reference, heterozygous, and homozygous alternate genotypes, respectively.

Our genome-wide *G*_ST_ and spatial ancestry analysis (SPA) scans identified genomic regions with significant population differences and spatial allele frequency patterns (Fig. 7A; Supplementary Data Table S4). The analysis detected 10 significant signals, of which 5 overlapped between both approaches (Supplementary Data Table S5). Additionally, our environmental genome-wide association (eGWAS) analyses detected 243 significant associations across the targeted panel linked to variables of elevation (*n* = 15), precipitation (*n* = 34), soil (*n* = 97) and temperature (*n* = 97) (Fig. 7A; Supplementary Data Table S6). The analysis showed that 42 genetic signals were shared between multiple environmental factors (Supplementary Data Table S5), with most overlaps between elevation and temperature variables and additional intersections between soil, precipitation, and temperature factors.

Although no loci overlapped across all approaches (Supplementary Data Table S5), we examined nearby regions in linkage disequilibrium (LD) to identify potential shared signals. Three notable cases were detected: on chromosome 5 between organic carbon density at 5–15 cm (Vmac_chr05_33388302) and SPA (Vmac_chr05_33395640; *r*^2^ = 0.63), on chromosome 6 between precipitation of the driest month (Vmac_chr06_33703561) and *G*_ST_ (Vmac_chr06_29258763; *r*^2^ = 0.43), and on chromosome 10 between temperature of the coldest quarter (Vmac_chr10_15584947) and *G*_ST_/SPA (Vmac_chr10_26646489; *r*^2^ = 0.57). We observed significant differences among genotypes for these three loci (Fig. 7B–D). In general, the reference homozygous genotypes (allele dosage = 0) were collected in areas with elevated organic carbon density at 5–15 cm soil depth, more precipitation during dry months, and warmer temperatures during the coldest season. The organic carbon density at 5–15 cm was significantly higher in individuals with allele dosage 0 (0.31 hg/m^3^) compared with heterozygotes (dosage 1 = 0.23 hg/m^3^) and alternate homozygotes (dosage 2 = 0.23 hg/m^3^) at *P*-value < 0.0001 (Fig. 7B). Individuals homozygous for the reference allele received 75.6 mm of precipitation during their driest month, while heterozygotes received 43.3 mm and alternate homozygotes received 28.4 mm (all *P*-values < 0.0001; Fig. 7C). The reference homozygotes were associated with the highest temperature values (−0.13°C) during the coldest quarter compared with the heterozygotes (−0.95°C) and the alternate homozygotes (−1°C) at *P*-value < 0.0001 (Fig. 7D). We noticed that the MN and WI populations contained mainly heterozygous and alternate homozygous genotypes (allele dosage = 1–2) while the Eastern populations were mainly reference homozygous (allele dosage = 0). Consistently, these loci found within (Supplementary Data Fig. S10) and (Supplementary Data Table S5) displayed clear allele distribution patterns across geographic regions. The Midwestern populations (MN and WI) exhibited reference allele frequencies between 0.08 and 0.43, whereas substantially higher frequencies (0.75–1.00) were observed in the Eastern populations. The MI population showed reference allele frequencies ranging from 0.40 to 1 (Supplementary Data Fig. S10).

To place these geographically differentiated wild alleles in an applied context, we examined allele frequencies at these loci in 484 genotypes derived from controlled crosses between 1996 and 2017 within the Rutgers University cranberry breeding program (Supplementary Data Table S1). We noticed that the Rutgers material exhibited high reference allele frequencies (0.72–0.90) within the range observed in Eastern wild populations (Supplementary Data Fig. S10) for several loci that exhibited West–East allele-frequency differentiation in wild populations (Supplementary Data Table S6). However, we did not observe a single homogeneous cluster for the breeding material in the principal component space (Supplementary Data Fig. S11), reflecting the population structure within the breeding program. Here, although many Rutgers genotypes overlapped with wild populations, several pedigree-driven populations were displaced along the main principal components and clearly separated from wild populations. This structure aligned with allele-frequency variation in a subset of loci exhibiting strong West–East differentiation in wild populations, where some pedigree groups displayed allele-frequency patterns similar to those observed in wild populations of the Midwest. For example, the breeding cluster that included CNJ08-233, CNJ12-56, and CNJ12-58 populations and located in the upper-left region of the PCA plot (Supplementary Data Fig. S11; Supplementary Data Table S1) showed a reference-allele frequency of 0.31 in Vmac_chr08_18499418, closely matching the frequency observed in wild populations in the Midwest (≈0.22) and contrasting with the frequency observed in wild populations in the Eastern region (≈0.70) and in other Rutgers subpanels. Similarly, CNJ16-41 population, which clustered with wild populations (Supplementary Data Fig. S11; Supplementary Data Table S1), exhibited a reference allele frequency of 0.25 in Vmac_chr09_22406509, comparable to MN–WI (≈0.19) and markedly lower than the frequencies observed in Eastern wild populations and other breeding clusters (≥0.70).

### Candidate gene annotation and predicted genomic effects of environmentally associated SNPs

We identified 839 candidate genes within ±17 kb of the top SNPs associated with environmental factors (Supplementary Data Tables S6 and S7), of which 442 showed putative homology to *Arabidopsis thaliana*. After removing duplicate Arabidopsis Gene Identifiers (AGIs), 406 unique AGI homologs were retained for Gene Ontology (GO) enrichment analysis. Of these, 165 AGIs were associated with temperature variables, 52 with precipitation, 170 with soil-related variables, and 32 with geographic variables. We detected only significant enrichment of GO with a false discovery rate (FDR) ≤ 0.05 for cellular component terms associated with temperature (Supplementary Data Fig. S12). The terms of enriched cellular components were primarily related to the components of the vesicle and endomembrane system, including intracellular and cytoplasmic vesicles and the endoplasmic reticulum. Among the temperature-associated loci that contributed to the enrichment of GO terms from cellular components, several candidate genes were notable for their functional annotations (Supplementary Data Table S7). Vmac_010611 (AT2G36620; 86.6% amino acid (aa) identity), ribosomal protein L24A (RPL24A), was associated with mean temperature of the driest quarter (Vmac_chr02_9861548) and temperature seasonality (Vmac_chr02_9865039). Vmac_021589 (AT1G59820; 76.3% aa identity), aminophospholipid ATPase 3 (ALA3), corresponded to temperature seasonality (Vmac_chr04_24969180). Additional temperature-related candidates included Vmac_033020 (AT5G05170; 85.5% aa identity), a cellulose synthase family protein (CESA3), for mean temperature of the wettest quarter (Vmac_chr07_7693613), and Vmac_038147 (AT1G70940; 76.9% aa identity), an auxin efflux carrier protein (PIN3), for minimum temperature of the coldest month and mean temperature of the coldest quarter (Vmac_chr08_14024051). Although no significant GO term over-representation was detected for precipitation, soil, or geographic variables, we identified several here as targets for future investigation. For organic carbon density (Vmac_chr05_33388302), notable candidates included Vmac_026877 (AT4G26270; 77.2% aa identity), ATP-dependent 6-phosphofructokinase 3 (PFK3), and Vmac_026876 (AT1G51965; 64.8% aa identity), ABA overly-sensitive 5 (ABO5). For precipitation-related variables (Vmac_chr06_33703561), candidate genes included Vmac_031275 (AT3G14750; 55.2% aa identity), FLX-like 1 (FLXL1), and Vmac_031273 (AT3G14770; 68.3% aa identity), a sugar transporter (SWEET2). For elevation and precipitation of the driest quarter (Vmac_chr09_22406509), Vmac_043829 (AT1G11930; 74.6% aa identity), a pyridoxal phosphate homeostasis protein (PLPHP1), was identified among five candidates within the interval.

We examined the predicted impact of the top SNPs on genomic features to identify their potential genomic effects (Supplementary Data Table S6). Of the lead SNPs, 50, 48, 85, and 3 were in the intergenic, exonic, intronic, and splice-site regions, respectively. Most SNPs were classified as MODIFIER impact (*n* = 134), followed by LOW (*n* = 29) and MODERATE (*n* = 23). The MODIFIER impact SNPs that were found in intronic regions included RPL24A (Vmac_chr02_9861548) and three other genes, called PFK3 (Vmac_chr05_33388302), FLXL1 (Vmac_chr06_33703561), and PLPHP1 (Vmac_chr09_22406509). By contrast, exonic SNPs showed a LOW or MODERATE impact, including ALA3 (Vmac_chr04_24969180), CESA3 (Vmac_chr07_7693613), and PIN3 (Vmac_chr08_14024051).

## Discussion

### Hidden rare-allele richness within wild cranberry populations

We noticed that the power to detect polymorphisms in wild cranberry populations varied in part due to uneven sampling, where the frequency distribution of common variants (MAF > 0.5) remained similar between populations, but rare alleles (0.01 ≤ MAF < 0.05) showed larger differences (Table 1). The observed heterogeneity was expected because the discovery of rare alleles below the frequency of 5% requires larger population sizes than the discovery of common variants [33–36]. For example, the two largest populations, MA (*n* = 45) and WI (*n* = 44), harbored 6.1–11.6 times more rare variants than the two smallest populations in this study (Table 1), the ME (*n* = 5) and NB (*n* = 4) populations. The Flex-Seq cranberry panel of 17 502 loci targets variants from core and accessory genes and known quantitative trait loci (QTLs), together with a smaller proportion of intergenic regions, derived from the *Vaccinium* pangenome [37, 38]. By design, this technology captures moderate frequency SNPs that are highly transferable between populations [38], which might explain why most of the variants detected in this study were common and overlapped widely between wild cranberry populations (Table 1, Fig. 1).

Although the distribution of rare variants between wild cranberry populations might have been restricted by uneven sampling, these patterns have been observed in other germplasm collections [39–42] that demonstrate how demographic history, clonal propagation, and local selection influence the rare tail of the allele frequency spectrum. Interestingly, half of the wild cranberry populations carried >50% rare private alleles (Fig. 1), which aligns with previous findings on cranberry population-specific allele enrichment at multiple hierarchical levels, including between cranberry species, within wild *V. macrocarpon* populations, and between wild and cultivated populations [23–25]. For example, Zalapa *et al.* [23] found that wild *V. macrocarpon* populations contained 2.4 times more private alleles than cultivated genotypes. Similar patterns have also been observed in other plant species, where environmental heterogeneity, geographic barriers, and local adaptation have been implicated in the accumulation of rare and private alleles in wild populations [43–45]. For example, genotype–environment association analyses in European beech (*Fagus sylvatica* L.) populations in Italy, Slovenia, and Croatia revealed SNPs linked to climate-responsive genes and site-specific allelic variants in heterogeneous landscapes [46], highlighting the adaptive potential of rare variants. Thus, in this context, the private allele richness of CWRs represents genetic uniqueness that should be prioritized in germplasm repositories for future cranberry cultivar improvement.

### Genetic erosion challenges local resilience across wild cranberry populations

We found *V. macrocarpon* exhibits exceptionally low nucleotide diversity (Fig. 2A), two to four orders of magnitude lower than the values observed in other outcrossing fruit crops [47–53]. For example, Manzanero *et al.* [53] reported *π* values from 1.2 × 10^−2^ to 2.3 × 10^−2^ in five blueberry species, while we observed an overall nucleotide diversity of 5 × 10^−6^ ± 2.2 × 10^−6^. Furthermore, the positive Tajima’s *D* value (*D̅* = 0.89; Fig. 2B) revealed an excess of intermediate frequency alleles (>0.20), with many rare alleles (<0.05; Supplementary Data Fig. S1). Differences in allele frequency composition across populations resulted in corresponding variation in *π* and Tajima’s *D* estimates. Populations with an excess of rare SNPs (e.g. MA and WI) showed lower *π* and Tajima’s *D* estimates, whereas populations with a deficit of rare alleles maintained greater diversity and deviation from neutrality (Table 1, Fig. 2A and B). The same inverse relationship has been observed in maize [54], papaya [55], and wheat [56], where rare alleles contribute little to *π*. Interpreting these *π* and Tajima’s *D* values requires consideration of biological processes shaping allele frequencies and the particular features of the targeted genotyping platform used here. From an evolutionary perspective, these statistics may reflect how balance of selection, population subdivision, or past demographic changes have impacted genomic regions [57, 59–62]. By contrast, similar patterns might also arise from the determination and filtering of SNPs associated with targeted genotyping strategies [58, 63]. Consequently, the *π* and Tajima’s *D* estimates in this study primarily capture diversity signals within the genomic regions targeted by the Flex-Seq panel, rather than representing unbiased genome-wide averages, and may under-represent variation in neutrally evolving intergenic regions. Targeted genotyping panels are preferentially designed to sample conserved or functional genomic regions, where *π* and Tajima’s *D* estimates derived from these approaches are expected to be lower than those obtained from whole-genome resequencing or restriction-enzyme-based reduced-representation methods due to the action of purification and linked selection in genic regions [58, 64]. In our study, MAF ≥ 0.01 SNPs were widely distributed throughout the genome (Supplementary Data Fig. S2) and exhibited dense marker spacing, and ≈65% of variants overlapped annotated gene models. These features highlight the utility of a targeted genotyping platform for generating dense and reliable data on important genomic regions. Furthermore, comparative studies have shown that, despite differences in locus composition and sensitivity to selection, sequence capture approaches can yield population-genetic summary statistics broadly consistent with those from restriction site-associated DNA sequencing (RAD-seq) methods in shallow evolutionary contexts. At the same time, capture-based methods provide improved locus consistency and orthology across populations [58, 63]. Consequently, *π* and Tajima’s *D* estimates reported here should be interpreted as a conservative lower bound on genome-wide nucleotide diversity and as mainly informative for relative comparison among cranberry populations within this targeted genomic panel.

Previous studies have shown contrasting results for genetic diversity using heterozygosity estimates [20–25]. Early allozyme work showed extremely low genetic diversity [21], while SSR and high-density SNP genetic studies revealed moderate to high levels of heterozygosity in cranberry populations [20, 22–25]. Our SNP dataset showed moderate levels of heterozygosity within and between populations (Table 1, Fig. 2C). Furthermore, we noticed that some populations (e.g. DE, MI, and PA) were more genetically uniform than others (e.g. ME, NB, and WI; Fig. 2C), similar to the patterns observed previously within populations [20, 21, 24, 25]. Inbreeding levels ranged from −0.13 (NB) to 0.15 (WI) at the population level (Table 1), while Bruederle *et al.* [21] indicated values between −0.82 and 1.00 and Rodríguez-Bonilla *et al.* [24, 25] indicated only negative *G*_IS_ coefficients. One explanation may be marker resolution, since those previous studies used alloenzymes and SSRs [65, 66], respectively. In contrast, our study used a pangenome-derived targeted genotyping platform to characterize variation in genic and QTL-linked regions, providing a more direct view of differentiation in functional genomic regions while maintaining marker consistency across populations. Another explanation might be the mating system among wild cranberry populations. For example, self-fertilization in cranberries produces a much lower seed set than cross-pollination [67], but this process might increase in areas with limited pollinator activity, such as WI [68]. Together, our results suggest that nucleotide diversity within the targeted Flex-Seq loci is exceptionally low, with population-specific shifts in allele frequency spectra, neutral equilibrium expectations, and inbreeding across the sampled range. The observation that approximately one-third of the accessions examined were heterozygous for at least one SNP unifies previous findings that wild cranberry maintains genetic variation that can be exploited in breeding programs. Thus, this analysis not only showed low nucleotide diversity, but also characterized diversity, Tajima’s *D*, and allele-frequency spectra across functionally enriched cranberry loci.

### Midwest–East genetic stratification and local admixture patterns in wild cranberry

Our study revealed moderate genetic differentiation in wild cranberry (*G*_ST_ = 0.14), which is consistent with the SNP-based estimate of *G*_ST_ reported by Neyhart *et al.* [20] but lower than those indicated by Bruederle *et al.* [21] and Rodríguez-Bonilla *et al.* [24, 25] using alloenzyme and SSR markers (*G*_ST_ = 0.22–0.33), respectively. The discrepancy of higher values of *G*_ST_ in these studies was probably due to a small number of markers that could have inflated their estimates of *G*_ST_. Interestingly, all the patterns observed from the pairwise comparisons of *G*_ST_ (Fig. 3A), MDS (Fig. 3B), PCA (Fig. 4) and our phylogenetic tree (Supplementary Data Fig. S6) converged on a pronounced geographically genetic stratification among populations. The MN and WI populations consistently formed a single upper Midwest cluster in all these analyses, while the Eastern samples maintained their own group. Neyhart *et al.* [20] and Rodríguez-Bonilla *et al.* [25] also observed a similar Midwest–East discontinuity, supporting the major population-structure and phylogenetic patterns recovered here under a targeted genotyping approach. However, some exceptions were observed. For example, the ME population exhibited the highest genetic divergence (Fig. 3A) yet remained part of the Atlantic block (Figs 3B and 5B); its elevated *G*_ST_ value could result from its heterogeneity patterns of the genome-wide Tajima’s *D* distribution (Fig. 2B) and the heterozygosity observed within the population (Fig. 2C). The MI population showed mixed results among analyses, as its *G*_ST_ values were lower relative to the Eastern populations (Fig. 3A) and grouped with the Eastern populations in MDS1 (Fig. 3B), while occupying an intermediate position between the Midwest and Eastern populations in PC1 (Fig. 4) and the phylogenetic tree (Supplementary Data Fig. S6). Neyhart *et al.* [20] found that the MI population was grouped with Eastern accessions, although their diversity panel did not contain MN accessions to clarify this separation. These results may suggest that MI is a transition zone between the two regions that could potentially serve as a gene flow corridor. Rodríguez-Bonilla *et al.* [25] showed that several genotypes split neatly into these two distinct groups, but some accessions created transitional groups that might connect the Midwest and Eastern regions. The complex genetic dynamics underlying the demographic history of wild cranberry populations became evident through analysis of admixture (Fig. 5A) and gene flow (Fig. 5B). The Midwest–East population exhibited separate ancestral groups, but the MI and WI populations suggest historical connectivity between the two regions. Interestingly, the MA population showed nearly pure C3 ancestry along with its wide distribution pattern observed in PC2 and the state-level genetic diversity found by Neyhart *et al.* [20]. Such geography-bound ancestry mosaics are common in plants [69–72] because their genetic makeup develops over time from historical range movements linked to seed dispersal and human activities that reconnect previously isolated gene pools.

### Late-glacial climatic change may have contributed to bottleneck and isolation in *Vaccinium macrocarpon*

Demographic inference based on the site frequency spectrum (SFS) is sensitive to sampling design, SNP ascertainment, and locus composition [73–75]. Because the targeted panel used here is enriched for genic and QTL-linked regions, purifying and linked selection may influence the SFS and bias absolute demographic parameter estimates [64, 76]. However, empirical and simulation-based studies show that targeted and reduced-representation datasets can yield conservative but informative estimates of relative population history, including divergence patterns, comparative effective population sizes, and migration signals [30, 58, 74, 76]. Additionally, absolute estimates of *N*_e_ and calendar time depend on the assumptions of mutation rate and generation time. Here, we applied a mutation rate of 6 × 10^−8^ substitutions per site per generation, obtained by converting a commonly applied dicot synonymous substitution rate assuming a generation time of 4 years for cranberry [77, 78]. Lower mutation-rate assumptions were also used in a previous demographic modeling study of lingonberry (*Vaccinium vitis-idaea* L.) [79]; such alternatives would proportionally rescale absolute demographic inferences but are less likely to affect the relative support for alternative demographic models or the inferred directionality of asymmetric gene flow, as these patterns are primarily driven by the shape of the SFS [80]. Consequently, our simulations provide a useful framework for interpreting the relative demographic history between Midwestern and Eastern cranberry populations.

The demographic inference of the genetic divergence between the Midwest and Eastern populations indicated that the two best-supported models (I and M) were nearly identical (Table 2, Supplementary Data Fig. S8), differing only in the inclusion of an ancestral bottleneck (Fig. 6). The minimal difference between these models (*Δ*AIC = 1) suggests that they differ only slightly in structure [81]. Although Model I was 2.1 times more probable than Model M, its weight (*ω* = 0.68_)_ remained below the 0.90 threshold to justify a single model [82, 83]. Therefore, we averaged these two models to account for model uncertainty and reduce potential bias in parameter interpretation. This approach balances fit and parsimony while incorporating evidence for a possible ancestral bottleneck, thereby providing a coherent explanation for the Midwest–Eastern divergence in wild cranberry populations. The demographic multimodel averaging suggests that the ancestral *V. macrocarpon* population went through a severe bottleneck between ≈15.9 ± 2.88 and 14.4 ± 0.23 kya (Fig. 6), a period that overlaps broadly with the rapid retreat of the Laurentide Ice Sheet across northern areas (e.g. MN, WI, and MI) and abrupt warming during the Bølling–Allerød Interstadial ≈14.7 kya [84, 85]. Although this temporal overlap does not by itself establish causality, it is consistent with paleoclimatic evidence for major environmental change during that interval, which may have reduced suitable habitat for the ancestral *V. macrocarpon* populations through changes in hydrology, vegetation, and peatland development in newly deglaciated landscapes. Wild cranberry grows mainly in acidic peatlands that are waterlogged – specifically in kettle-hole bogs developed in postglacial depressions above coarse outwash sand [25, 86]. The newly deglaciated Glacier Bay lakes in Alaska initially carried high pH, alkalinity, and salinity of the waters that progressively decreased thereafter [87]. Furthermore, a study of radiocarbon-dated peatlands indicated that a few peatlands were initially formed near retreating ice margins between 16.2 and 14.7 kya, when the deglaciation had advanced from 12 to 20% [88]. The lack of suitable basins and newly dry deglaciated terrains limited the development of peatlands [88], the few sites that formed accumulated peat at only steady and low long-term rates [89], and as a result peat carbon sequestration remained essentially negligible ≈15 kya [90]. The first appearances of *V. macrocarpon* and other peatland species, such as *V. oxycoccos* and *Sphagnum* spp., have been reported after ≈14 kya [91–94], suggesting that cranberry likely re-expanded from a handful of ice-free southern North American refugial peatlands that may have sheltered the ancestral populations after the bottleneck. A previous study of wild cranberry populations, using 23 allozyme loci and Nei’s distance-based calculation, dated a severe Pleistocene bottleneck to ≈110 kya [21]. The discrepancy likely reflects differences in marker choice and methodology [65, 80, 95], but collectively these results might suggest that *V. macrocarpon* has experienced more than one bottleneck.

The Huron–Michigan–Superior and Ontario basins maintained proglacial waters at our inferred Midwest–Eastern split time (≈12.7 ± 0.11 kya; Fig. 6), forming Glacial Lake Algonquin and late Lake Iroquois phases [96–98]. These events could have facilitated the northward spread of cranberry from southern refugial areas along the margins of proglacial lakes and meltwater outwash plains, which functioned as migration corridors and as hydrological barriers that contributed to the formation of distinct Midwestern and Eastern groups. The 1680 basal-peat dates indicated that peatland formation was dense along the Atlantic coast to New Brunswick but remained sparse west of Lake Superior at ≈12 kya [88]. Therefore, the deglacial period may have maintained separation between the Eastern and Midwestern cranberry ancestors for ≈11.4 kyr, with suitable habitat remaining scattered in the Midwest while a more continuous Atlantic bog corridor developed earlier in the East. As the Holocene period progressed, North American peat core records indicated that peatlands expanded while becoming more hydrologically mature [99–102]; by ≈1.3 ± 0.09 kya (Fig. 6) this expansion could have allowed gene flow between Midwestern and Eastern populations. The introgression analysis at the river basin level (Supplementary Data Fig. S8, Supplementary Data Table S3) revealed that the gene flow was most extensive among rivers within the Eastern region, followed by significant introgression within the Midwest and limited genetic exchange between rivers of the two regions. These patterns likely reflect geographic proximity and postglacial hydrological connections that facilitated gene flow among cranberry populations. Similarly, Reusch *et al.* [103] showed that the genetic structure of stickleback populations was shaped not only by geographic distance but also by postglacial hydrological patterns and habitat heterogeneity, highlighting the role of water bodies in maintaining connectivity between populations. River-associated gene flow in cranberry may also represent a broader postglacial dynamic in which water bodies facilitated range expansion and maintained connectivity among populations. Although hydrological pathways may have played a major dispersal role, other mechanisms may also have contributed, including aquatic and small birds for long-distance movement, as well as mammals, local water flow, or human-mediated transport of cranberry propagules (seeds, cuttings, peat mats) over shorter distances [104–106]. Consequently, asymmetric migration (0.02 ± 0.18 diploids per generation from the Midwest to the East and 2.2 ± 0.23 in the opposite direction), together with higher admixture (Fig. 5A) and 3.7-fold greater *N*_e_ in the Eastern population, suggest that the larger and more connected Eastern population likely acted as a source of westward gene flow (Fig. 5B). Similar regional patterns of asymmetric gene flow have been documented in North American conifers such as black spruce (*Picea mariana*) and red spruce (*P. rubens*) following postglacial recolonization, supporting the idea that climatic oscillations and ecosystem shifts contributed to these dynamics [107]. These findings suggest that genetic differentiation in wild cranberry was shaped by habitat changes during the late glacial period and by subsequent hydrological expansion during the Holocene. These interpretations should be viewed as paleogeographic hypotheses that fit with the demographic results rather than uniquely identified historical scenarios. However, this study provides the first formal evaluation of alternative demographic scenarios for wild cranberry populations, distinguishing between a split-with-gene-flow model and a closely related model that includes an ancestral bottleneck. Furthermore, the absence of *V. macrocarpon* accessions from the Pacific Northwest limits our ability to fully reconstruct the historical processes that shaped the present-day genetic diversity observed in wild cranberry populations. Rodríguez-Bonilla *et al.* [25] identified a genetically diverse wild population in the Okanogan–Wenatchee National Forest, Washington State, that clustered separately from Eastern and Midwestern germplasm and exhibited several private alleles. These observations underscore the importance of including Western populations in future demographic studies to uncover how *V. macrocarpon* populations evolved through glacial refugia and postglacial migration paths.

### Environmental adaptation and allele enrichment across wild and breeding germplasm

Our findings align with and build on those of Neyhart *et al.* [20], who reported environmental associations among wild cranberry populations linked to soil texture, geography, precipitation, and temperature gradients. Several of our loci were located within <50 kb of signals identified by this previous study, where we found four distinct chromosomal segments which appeared as adaptive regions in both studies. Our elevation locus on chromosome 1 is close to their soil-related factors within an 11-kb range. The temperature-seasonality signal on chromosome 4 coincides within a 6-kb interval identified previously. Additionally, we noticed overlapping soil-related loci on chromosome 6 that span from 12 to 15 kb and on chromosome 10 <50 kb away between both studies. Interestingly, chromosome 6 stands out because our *G*_ST_/precipitation of the driest month locus is located near their mean temperature of the driest quarter signal, which is <4 kb from our *G*_ST_ locus, suggesting a shared climatic driver linked to adaptation under dry-season conditions. Consistent with their observation that minor allele frequencies appeared more frequently in areas with cold and dry conditions, our results showed that Midwestern populations with lower temperatures and reduced precipitation had primarily heterozygous and alternate homozygous genotypes (allele dose = 1–2). By contrast, Eastern populations consisted mainly of reference homozygotes (dosage = 0; Fig. 7B–D, Supplementary Data Fig. S10). The parallel patterns between our study and Neyhart *et al.* [20] support the presence of geographically structured candidate loci associated with environmental gradients among wild cranberry populations.

The North American cranberry was domesticated during the early 1800s through the selection of native genotypes from wild populations collected in MA, MI, NJ, and WI [108, 109]. The Rutgers program was revived in 1985 through the collection of wild germplasm, which led to the development of second-generation hybrids, including ‘Crimson Queen’, ‘Demoranville’, ‘Mullica Queen’, ‘Scarlet Knight’, ‘Welker’, and ‘Haines’, derived from first-generation hybrids and native selections. We observed that the 484 Rutgers-derived cranberry genotypes are enriched for reference alleles at the Western–Eastern pattern loci, generally within the range observed in Eastern wild populations (Supplementary Data Fig. S10). This bias may reflect, at least in part, the initial source of the founding material, the breeding history of the program, and the environmental conditions of NJ bogs. However, our PCA, including both wild and Rutgers materials (Supplementary Data Fig. S11, Supplementary Data Table S1), showed that the Rutgers breeding panel is not genetically homogeneous but instead comprises pedigree-defined subpanels that align differently along the central axis of the wild population structure. In long-lived perennial crops, such a persistent internal population structure is expected because relatively few sexual generations and repeated recycling of elite parents can maintain strong pedigree stratification and non-random allele-frequency differences between breeding subgroups [1, 110]. Although directional selection during breeding is a plausible contributor [18, 20], the present analyses do not distinguish selection from founder effects or pedigree structure. Midwestern allele-frequency states at a subset of loci were observed in specific Rutgers families rather than distributed throughout the breeding panel; for example, CNJ12-56, CNJ12-58, and CNJ08-233 exhibited Midwestern frequencies at Vmac_chr08_18499418, whereas similarly low Midwestern frequencies at Vmac_chr09_22406509 were detected only in families grouped with wild populations and not in other Rutgers subpanels. This analysis adds an applied dimension to the environmental-association results by assessing whether geographically differentiated wild alleles with potential breeding value are already represented in breeding germplasm or remain underused. Therefore, targeted introgression of wild alleles from distinct geographic sources – guided by locus-specific patterns of allele representation, environmental association, and pedigree structure – may broaden the genetic diversity of elite breeding material and improve its capacity to respond to changing climates, emerging diseases, and marginal environments. Because our results are based on a single breeding program, the observed allele-frequency skew should not be assumed to represent all elite cranberry germplasm.

### Genome–environment associations reveal stress-responsive candidate genes

Genome–environment association analyses offer a valuable first step toward identifying genomic regions that may contribute to environmental responses in heterogeneous landscapes. Based on these associations, we used the top SNPs associated with environmental variables to identify candidate genes nearby within ±17 kb for further analysis (Supplementary Data Tables S6 and S7). We detected a significant over-representation of vesicle and endomembrane cellular components only between genes located in temperature-associated genomic regions (Supplementary Data Fig. S12), which are important in plant stress response through their ability to modify membrane structure and protein transport and cellular signal transmission during environmental changes [111, 112]. For example, RPL24A (Vmac_chr02_9861548; mean temperature of the driest quarter and temperature seasonality), has been implicated in responses to dehydration and osmotic stress in *A. thaliana* by increasing proline production and related physiological mechanisms during drought stress [113]. ALA3 (Vmac_chr04_24969180; temperature seasonality) has shown temperature-dependent growth and reproductive phenotypes consistent with its role as a trans-Golgi-associated P4-ATPase involved in endomembrane remodeling and acclimatization to variable growth environments [114]. CESA3 (Vmac_chr07_7693613; mean temperature of the wettest quarter) encodes a cellulose synthase subunit predominantly expressed in roots. The disruption of cellulose synthesis in roots has resulted in reduced cellulose content, abnormal root structure, and constitutive activation of jasmonate and ethylene signaling pathways for stress response [115–117]. PIN3 (Vmac_chr08_14024051; minimum temperature of the coldest month and mean temperature of the coldest quarter) mediates auxin transport in roots and exhibits impaired intracellular trafficking under cold temperatures and osmotic stress, causing altered auxin distribution, constrained root growth, and defects in gravitropic and hydropatterning responses [118–121]. These examples provide a biologically coherent subset of candidates that link temperature-dependent genome–environment interactions to experimentally characterized stress-responsive genes.

Although precipitation-, soil-, and geography-associated loci did not show significant GO enrichment, further exploration of candidate genes is worthwhile, as several exhibited distinct geographical patterns of allelic distribution (Supplementary Data Tables S6 and S7). This lack of significant enrichment may partly reflect the composition of the Flex-Seq panel, which is enriched for core genic regions and may favor broader functional categories, thereby reducing the power to detect more specific over-represented terms for other environmental variables. Nevertheless, the organic carbon density at Vmac_chr05_33388302 showed West–East allele-frequency differentiation (Fig. 7B). PFK3 was prioritized because it is strongly expressed in roots with root epidermal patterning, and induced under energy-limiting stresses (heat and oxygen deficiency), which is in line with root-level responses to heterogeneous soil environments [122–124]. Similarly, from the precipitation-associated locus Vmac_chr06_33703561 with West–East allele-frequency patterns (Fig. 7C), FLXL1 emerged as a plausible candidate. FLX-family genes regulate the FRIGIDA-FLOWERING LOCUS C (FRI-FLC) flowering pathway, which controls flowering time through FLC repression [125, 126, 127]. Because plants often adjust flowering time under water limitation as a drought-escape strategy, natural variation within the FRI–FLC regulatory module provides a biologically relevant mechanism during the critical water shortage period [128]. In contrast to loci showing broad West–East differentiation, the elevation-and precipitation-associated locus Vmac_chr09_22406509 displayed allele-frequency differences within breeding materials (e.g. CNJ16-41). PLPHP1 was identified as a strong candidate from the five genes within this genomic interval because it regulates pyridoxal phosphate levels and environmentally sensitive root development. Loss of PLPHP function causes severe root meristem defects and reduced root growth under specific environmental conditions [129], highlighting that this locus may reflect localized selective pressures acting on specific breeding lines under adverse environmental conditions. The loci analysed illustrate how environmental signals outside of temperature-enriched pathways can identify meaningful genetic candidates whose effects may be context-dependent and population-specific.

Several highlighted loci contained top SNPs in exonic regions (Supplementary Data Table S6), including PIN3 (MODERATE impact), CESA3, and ALA3 (LOW impact), which may influence protein function or stability. However, most lead SNPs were classified as MODIFIER and located in intronic or intergenic regions. Non-coding regions, including promoters and introns, are known to frequently harbor regulatory variation that affects transcription and splicing, and therefore can contribute to adaptive responses to climatic stress [130, 131]. Despite these genetic signals, candidate-gene selection represents a challenging step. Genome-wide association analyses often detect broad association peaks shaped by linkage disequilibrium, population structure, and polygenic effects, which may span large genomic regions and spurious associations [132, 133]. In this study, candidate genes were selected using proximity to lead SNPs and analysed for those with functional annotation available, largely based on homology to annotated *Arabidopsis* genes. Therefore, genes without AGI annotation or clear functional inference were not considered, which is a common limitation in non-model systems where incomplete annotation tends to lead researchers to focus on the interpretation of genes with existing functional information [134]. Further integration of fine-mapping, gene expression analyses under controlled environmental conditions, and functional validation will be necessary to identify causal variants and clarify their roles in stress-related phenotypes in cranberry.

### Conclusions

The genetic diversity present in wild cranberry populations represents a reservoir of alleles that may help breeding programs respond to environmental change. The patterns observed here also reflect the influence of past climatic and geographic events on the evolutionary history of *V. macrocarpon*. Although our results are broadly consistent with previous studies of population structure in wild cranberry, this study extends that foundation by providing the first targeted-panel estimates of nucleotide diversity and SFS-based demographic history in the species using a *Vaccinium* pangenome-derived sequencing platform. Because this platform is enriched for genic and QTL-linked regions, these inferences should be interpreted as conservative targeted-panel estimates rather than unbiased genome-wide summaries. Our findings further show that wild cranberry contains regionally structured allele distributions, including candidate environmentally associated variants that may be relevant to adaptation and useful for breeding. These results demonstrate how targeted genotyping approaches can complement other sequencing strategies by providing conservative but informative insights into population history and allelic differentiation in functionally important genomic regions of the genome. Overall, this work underscores the importance of conserving wild cranberry germplasm as a source of genetic variation that may contribute to future trait improvement under changing environmental conditions.

## Materials and methods

### Plant material

We studied 179 genotyped accessions of North American wild cranberry collected from natural populations (Supplementary Data Table S1), including 121 accessions maintained by Rutgers University [20] and 58 by the University of Wisconsin [24]. The accessions were collected from 37 different sites that covered nine US states (DE, MA, ME, MI, MN, NJ, NY, PA, and WI) and one Canadian province (NB). These locations represent the main distribution area of *V. macrocarpon* in North America [135], with sample sizes ranging from 1 to 36 accessions per site.

A subset of elite cranberry breeding material from the Rutgers University cranberry breeding program was analysed to contextualize the allele-frequency differentiation patterns observed in wild populations (Supplementary Data Table S1). The panel included 484 genotypes obtained through controlled crosses between 1996 and 2017 from 32 pedigree-defined families. The family sizes varied widely, ranging from 1 to 217 genotypes per family, due to uneven representation among genotyped and retained progeny available across families. These genotypes were genotyped using the same Flex-Seq targeted sequencing platform and quality-control pipeline applied to the wild accessions. The breeding material was analysed alongside wild populations using PCA and allele-frequency comparisons to determine the representation of geographically differentiated wild alleles in elite germplasm.

### DNA variant calling

Young leaf tissue was collected and dried with a Labconco Freezezone lyophilizer (Kansas City, MO). Genomic DNA was extracted from lyophilized tissues for library preparation and then sequenced with the high-density 17 K cranberry Flex-Seq targeted genotyping platform developed from the *Vaccinium* pangenome (FS_12902; clare2026high). The panel contains 17 502 loci distributed across the 12 cranberry chromosomes, where most of them reside in genic regions (core and accessory genes) and a smaller proportion in intergenic spaces. We identified SNP and INDEL DNA variants using sequences aligned with the reference genome ‘Ben Lear’ v1.0 [136]. The raw sequencing reads were trimmed with Cutadapt v2.6 [137] to remove adapter contamination and low-quality bases (−q 20 -m 30). After trimming, only samples with missing data ≤25% were retained for subsequent analyses. Quality-trimmed reads were mapped to the reference genome using the BWA 0.7.17 aligner tool [138]. The prediction of variant calls was performed with the GATK4 workflow [139]. BCFtools v1.20 [140] was used for post-call variant filtering to retain DNA variants with a minimum average read depth of 10 and missing data ≤10%. This variant call workflow was implemented throughout the collection and independently of each of the 10 state/province populations for specific downstream analysis (Supplementary Data S1). The physical distances between adjacent SNPs retained after filtering, along with their classification as genic or intergenic, were determined using the reference genome annotation.

### Genetic diversity and differentiation analyses

We used the view function from BCFtools v1.20 [140] to identify rare and common DNA variants across populations. DNA variants were filtered according to the MAF thresholds, where the rare and common alleles were defined as 0.01 ≤ MAF < 0.5 and MAF > 0.5, respectively. The intersection of variants among populations was analysed and visualized using the UpSetR R package [141].

Rare and common SNPs were only considered to analyse *π* and the neutrality test (Tajima’s *D*) within each population. SNPs were obtained through the BCFtools v1.20’s view function [140]. We estimated *π* and Tajima’s *D* with VCFtools v0.1.16 [143] using a sliding window of 100 000 bp and a step size of 50 000 bp. Finally, windows with ≤5 SNPs were excluded to reduce noise in estimates. The analysis of the heterozygosity estimates *G*_ST_ and *G_IS_* included only pruned common SNPs. The SNPs were pruned with PLINK 1.90 [142] to minimize redundancy, applying a sliding window of 50 SNPs, the step size of 5 SNPs and the threshold of *r*^2^ > 0.9. The –het function of VCFtools 0.1.16 [143] was used to calculate the individual heterozygosity of common SNPs specific to the population. The pairwise WCfst function in the hierfstat [144] R package was used to estimate pairwise *G*_ST_ following Weir and Cockerham [145]. We estimated *H*_O_, *H*_E_, and *G*_IS_ coefficients using the basic.stats function in hierfstat [144].

### Population structure and phylogenetic analyses

We analysed population stratification in the collection using PCA with pruned common SNPs (15 325 SNPs) using the prcomp function in R. Furthermore, we evaluated spatial patterns of genetic differentiation between collection sites by determining genetic distances. We calculated the pairwise distance using the genet.dist function in hierfstat [144] with the *G*_ST_ method [145]. A distance matrix was computed with the dist function, followed by a principal coordinate analysis through the cmdscale function in R. To detect admixture levels in all accessions studied in this research, pruned common SNPs were used in the ADMIXTURE v1.3 program [146]. The cross-validation (CV) error was recorded and plotted against cluster numbers (*K*) from 1 to 10. The optimal *K* was determined as the value at which the CV error became negligible based on ‘elbow’ heuristic analysis. The proportions of admixture at this optimal *K* were then visualized and interpreted as the best-supported clustering solution for the accessions studied.

To infer the phylogenetic relationships between wild populations, we built an individual-level tree using pruned common SNPs (MAF ≥ 0.05, *r*^2^ > 0.9). The missing genotypes were imputed with the mean for each locus using the function A.mat from the rrBLUP R package [147]. The Euclidean genetic distance between individuals was calculated using the dist function in R [148], and a neighbor-joining tree was constructed with the ape R package [149]. This topology served as the starting tree for maximum-likelihood optimization under an ordered substitution model with 1000 bootstrap replicates to assess node support, implemented in the phangorn R package [150]. The ggtree R package [151] was used to visualize the final phylogenetic tree.

### Population demography and gene flow

The demographic inference of wild cranberry populations in North America was performed using the fastsimcoal2 v2.8 program [80]. The observed joint SFS needed to run coalescent simulations was assembled with 30 818 rare and common SNPs through the easySFS v0.01 program [152]. Our approach was to calculate the observed SFS for the upper Midwest (MI, MN, WI) and Eastern (DE, MA, ME, NB, NJ, NY, PA) groups by projecting a sample size of 126 and 178 in a window size of 100 000 bp, respectively. We tested 14 demographic scenarios, including population bottleneck, divergence, growth rate, and migration (Supplementary Data Fig. S7). Each model was run 50 times with randomly initialized parameters to increase the chance of finding the maximum log-likelihood. A mutation rate of 1.5 × 10^−8^ per site per year reported for dicotyledonous plants was fixed [77, 78] and converted to 6.0 × 10^−8^ substitutions per site per generation assuming a 4-year life cycle for cranberry. Each replicate included 100 000 coalescent simulations, 40 iterations of the ECM (conditional expectation–maximization) algorithm, and restricted SFS entries using a minimum count threshold of 5. We calculated AIC scores [153] for the highest composite log-likelihood per model to identify the best-fit demographic model. Each top-scoring model was then re-simulated 100 times to approximate the likelihood distribution and evaluate overlap between model likelihood distributions to test for statistical significance. We used block-bootstrap replicates of the joint SFS to quantify parameter estimate uncertainty through easySFS [152] by generating 50 replicates of 100 independently resampled genomic segments (100 000 bp per segment). The two best-fit demographic models were re-optimized 100 times for each replicate in fastsimcoal2 [80] and the run with the highest log-likelihood was selected. The boot R package [154] processed the set of 50 maximum likelihood estimates to generate two-tailed 95% confidence intervals for each parameter. The parameters of the effective population size (*N*_e_) expressed in terms of gene copies were converted into diploid individuals. The time parameters as the number of generations were converted to years by multiplying a generation time of 4 years.

Genetic introgression signals between populations were evaluated with Dsuite v0.58 [155], using common SNPs and a neighbor-joining tree of 179 wild cranberry accessions rooted with two samples of *Vaccinium oxycoccos* as outgroup. The tree was simplified by collapsing clades so that individuals from the same population were represented as a single tip. The resulting tree topology was used to compute the *f*_4_ ratio and Patterson’s *D* statistics with the Dtrios function. Correlated *f*_4_ ratio estimates were summarized into branch-specific *f*_b_ metrics using the Fbranch function, and the results were visualized as a heat map generated with the dtools.py script.

### Genome-wide association analysis and candidate genes

Locus-specific *G*_ST_ values were calculated with the wc function in the heirfstat [144] R package to detect genome-wide signatures associated with population differentiation. Loci exceeding the 99.9th percentile of the *G*_ST_ distribution were identified as *G*_ST_ outliers. Furthermore, we performed a SPA analysis with the SPA v1.13 program [156] by modeling SNP allele frequencies based on geographic coordinates (latitude, longitude; Supplementary Data Table S1). Spatial outliers were detected with SPA scores above the 99.9th percentile of the genome-wide SPA score distribution.

We conducted a genome-wide scan for SNP markers associated with bioclimatic and soil variables using a linear mixed-model approach. Bioclimatic variables were downloaded from the WorldClim database [157], and soil data were obtained from the SoilGrids database [158], both at 2.5 minute resolution, using the approximate geographic coordinates of the sampling location for each of the cranberry populations (Supplementary Data Table S1). The general form of the mixed model was **y = Qw + Xβ + Zu + e**, where **y** is a vector of an environmental variable, **Q** the incidence matrix of the vector of fixed effects (**w**), **X** the SNP genotype matrix, **β** the effect of the tested SNPs, **Z** the incidence matrix of the vector of polygenic background effects (**u**), and **e** the vector of residuals. The additive genomic relationship matrix, **K**, was used to model the covariance of the effects of the polygenic background, thus accounting for the effect of the background SNPs and the population structure. For each bioclimatic/soil variable, eight different models were tested that included fixed terms to account for population structure (i.e. 0 or 1 principal components of the **K** matrix) and geography (i.e. latitude, longitude, or latitude + longitude). The models were fitted using the GWASpoly R package [159]. The ideal model was selected based on visual inspection of *Q*–*Q* plots and which resulted in a *P*-value inflation factor [160] closest to 1. Significant marker–environment associations were declared using an FDR of 0.10.

Candidate genes associated with environmental factors were identified by selecting annotated genes from the reference genome ‘Ben Lear’ v1.0 (https://www.vaccinium.org/; kawash2022benlear) located within ±17 kb of the lead SNPs [20]. Putative homologs were identified in *Arabidopsis thaliana* based on amino acid sequence identity, and only genes with assigned AGIs were analysed. GO enrichment analyses were conducted using the clusterProfiler [161] and org At.tair.db [162] R packages. Over-representation tests were conducted for each environmental category (temperature, precipitation, soil, and geographic variables) with all candidate genes annotated with AGIs as the background universe. Biological process, molecular function, and cellular component ontologies were analysed, and the Benjamini–Hochberg FDR correction method was used to determine statistical significance. The functional effects of lead SNPs were predicted using SnpEff v5.2c [163], and the variants were classified according to their genomic context and predicted impact on gene structure.

## Supplementary Material

Web_Material_uhag161

## Data Availability

Data associated with this study are publicly available in a Dryad repository (https://doi.org/10.5061/dryad.m0cfxpph5).
